# Comparison of substance use, subjective well-being and interpersonal relationships among young people in foster care and private households: a cross sectional analysis of the School Health Research Network survey in Wales

**DOI:** 10.1136/bmjopen-2016-014198

**Published:** 2017-02-20

**Authors:** Sara Jayne Long, Rhiannon E Evans, Adam Fletcher, Gillian Hewitt, Simon Murphy, Honor Young, Graham F Moore

**Affiliations:** DECIPHer, UKCRC Centre of Excellence, Cardiff University, Cardiff, UK

**Keywords:** PUBLIC HEALTH, wellbeing, alcohol, looked after children and young people, Social care

## Abstract

**Objective:**

To investigate the association of living in foster care (FC) with substance use and subjective well-being in a sample of secondary school students (11–16 years) in Wales in 2015/16, and to examine whether these associations are attenuated by the perceived quality of interpersonal relationships.

**Design:**

Cross-sectional, population-based health behaviour and lifestyle questionnaire.

**Setting and participants:**

Wales, UK; young people who took part in the 2015/16 School Health Research Network (SHRN) questionnaire (n=32 479).

**Primary outcome:**

Health behaviours among young people in FC were compared with those from private households.

**Results:**

The prevalence of all adverse outcomes was higher among young people in FC. Those in FC were significantly more likely to report mephedrone use (OR=9.24, 95% CI 5.60 to 15.34), multiple substance misuse behaviours (OR=3.72, 95% CI 2.30 to 6.00), poorer relationships with peers (RR=1.88, 95% CI 1.23 to 2.88) and teachers (RR=1.83, 95% CI 1.31 to 2.56), having experienced bullying (OR=1.80, 95% CI 1.38 to 2.35), dating violence (OR=1.66, 95% CI 1.13 to 2.43) and poor well-being (RR=1.72, 95% CI 1.20 to 2.46). The association between FC and substance use remained significant, though was attenuated after accounting for relationship variables. The association between FC and subjective well-being became non-significant after adjustment for relationship variables.

**Conclusions:**

Young people living in FC experience significantly worse outcomes than young people not in care, likely due to a range of care and precare factors, which impact adversely on subsequent social relationships. The analyses are consistent with the hypothesis that the associations of FC with substance use and life satisfaction are partially explained by poorer quality social relationships. Large scale, longitudinal studies are required to investigate the relationship between being in care and health, educational and social outcomes. Mental health interventions and interventions to reduce substance use and improve well-being in FC should include a focus on supporting healthy social relationships.

**Strengths and limitations of this study**

- This study included a large sample size, which facilitated a robust modelling approach.
- School-level self-report data are not sufficient for understanding the trajectories of children. Strategic plans for data linkage would facilitate an understanding of the long-term impacts of health behaviours on outcomes.
- This study highlights a need for development and evaluation of interventions to improve outcomes among children in care, specifically those targeting relationship formation and maintenance.
- This cross-sectional design does not identify cause and effect.

## Introduction

In 2015, there were around 1.8%^1^ (5615) young people in Wales in local authority care, 50% higher than the English prevalence for the same period (1.2%). Otherwise known as looked after young people (LAYP), approximately three quarters of these young people in care were in foster care (FC). Studies from the UK^2–4^ and beyond^5–7^ indicate that LAYP have poorer health and educational outcomes than young people not in care, including higher rates of substance use, poorer mental health and lower well-being.^8–13^ Care-related factors, including placement type, periods in care and number of placements^14^ have been found to predict a range of health outcomes.^15^ ^16^ However, there is also evidence that FC can be protective. Educational outcomes have to date received more attention than health outcomes, with limited evidence regarding the role of the care system in shaping adverse outcomes.^17–19^ In one study, for example, examination pass rates among LAYP in care for over five years were twice as high as for LAYP who had been in care for only 1–1.5 years.^20^ Hence, while limited, there is evidence that FC can be associated with poorer, or better, outcomes. Nevertheless, when comparing LAYP with general population samples of young people living with birth parents, consistently poorer outcomes are observed across international contexts, despite diversity in care systems.^21^ Health or educational outcomes are perhaps not determined solely by care; universal processes associated with being in care, or preceding entry to care, are likely to contribute substantially to poorer outcomes.

A range of precare factors have been shown to be more common among young people who enter care than those who do not. A 2011 review^22^ highlighted a wide range of parental factors (socioeconomic status (SES), maternal age at birth, learning difficulties, ethnicity, single parenthood, smoking in pregnancy, mental illness and alcohol misuse) and child factors (low birth weight, prematurity, disability and attendance at accident and emergency departments) associated with entry to care. Histories of maltreatment are strong predictors of entry to care in a range of international contexts.^23^ ^24^ Indeed, in the 2015 Children in Need (CIN) census in Wales^25^ primary reasons for referral to social services included adverse childhood experiences (ACEs) such as domestic abuse (21%), parental substance use (18%) and parental mental health (15%), while among LAYP, primary needs related to abuse and neglect (66%), family dysfunction (14%) and acute family stress (7%).

Many of these adverse experiences, which are disproportionately experienced by LAYP, are linked to poorer health outcomes^26–32^ perhaps in part via their effects on social relationships. Early neglect can be harmful for children's socioemotional development.^33^ ^34^ Chronic fear and hyper-arousal as a result of abuse can cause the release of excessive cortisol, which has lasting effects on brain development,^33^ ^35^ impairing ability to regulate emotions, leading to poorer classroom performance, high emotional reactivity and poorer memory and retention.^33^ While healthy social relationships can protect against ill mental health and substance abuse,^36–41^ adverse relationships with caregivers can inhibit formation of subsequent healthy relationships.^42–47^ Young people who experience adversity,^48^ and LAYP^49^ ^50^ are more likely to present attachment disorders^51^ and to experience victimisation and bullying by peers^52^ in the school setting. Adverse home environments have been associated with increased risk of becoming a perpetrator and victim of violence,^52^ ^53^ including dating and relationship violence.^54–56^Further, the stigma associated with membership of a marginalised social group^57^ may compound relationship difficulties, leading to victimisation and poorer treatment by teachers and peers.^53^ Effects of the quality of relationships with peers and teachers on health and well-being have been recognised;^58–60^ and poorer relationships have been associated with substance use, antisocial behaviour and poorer mental health.^61–65^ Hence, a range of psycho-social and sociological processes associated with experiences leading to foster care, and the experience of being a LAYP, may impact negatively on the formation of health-protective relationships, potentially increasing the risk of substance use and poorer emotional well-being.

This paper draws on a large school-based survey of the health and well-being of young people aged 11–16 in Wales. First, we examine associations of living in FC with substance use (smoking, cannabis use, binge drinking and use of novel psycho-active substances), subjective well-being and relationships with teachers, peers and romantic partners. Subsequently, we test the hypothesis that young people in FC will report poorer relationships with peers and teachers, and will be more likely to report experiences of bullying and dating violence. Finally, we examine the extent to which the associations of living in FC with substance use and poorer emotional well-being are attenuated by differences in the quality of social relationships.

## Methods

### Study design and recruitment

This study uses data collected from the School Health Research Network (SHRN) student health and well-being survey in Wales in 2015. At the time of the survey, network schools represented just over half (N=113; 53%) of all secondary schools in Wales (N=212), with representation in all 22 local authority areas. Schools were recruited to the network through three mechanisms. First, those participating in the Welsh Health Behaviour in School-aged Children (HBSC) survey in 2013/14^66^ were invited to join (60 out of 82 secondary schools approached joined the network). Second, nine schools in South Wales that were recruited to a HBSC substudy in 2013 to pilot data linkage methods joined the network. Finally, 44 schools joined in 2015 during a period of open recruitment. Each member school had a designated member of staff who acted as a contact person and they were briefed about the survey via emails, newsletters and at an event for schools in June 2015. All network schools were invited to participate in a cross-sectional survey of students between September and December 2015. The survey was an online, closed-response, self-completion survey, available in English and Welsh. The survey was piloted with nine students (five girls) from years 7–10. Students went through the survey questions individually and then discussed their clarity and acceptability with a researcher. Minor changes to some items were made as a result of the piloting. Schools managed survey implementation using their own IT facilities. Schools were asked to include all students, but if not possible, to include a minimum of two mixed ability classes per year. Schools were asked to oversee students taking the survey. Staff were asked to remain at the front of the room unless a student asked for help. Schools could opt out of the drug-related questions if they chose to.

### Measures

Questions on the use of mephedrone were adapted from the drugs questionnaire used in the Avon Longitudinal Study of Parents and Children (ALSPAC).^67^ All other questions on substance misuse, subjective well-being and relationships with teachers and peers were adapted from the HBSC survey in 2013/14,^66^ a cross-sectional, international school-based survey developed by the WHO to understand young people's well-being, health behaviours and social context. Questions on romantic relationships and dating and relationship violence were adapted from Barter *et al*^68^ and the Revised Conflict Tactics Scale (CTS2).^69^

### Living arrangements

Students were asked who they lived with, with options of mother, father, stepmother, stepfather, foster mother, foster father or other. For the purposes of these analyses, a ‘living arrangements’ variable was created whereby students were classified as living with both parents, a single mother, a single father, in a stepfamily or in foster arrangements. Young people who lived in other unspecified arrangements were excluded from analysis.

### Frequency of current smoking

Frequency of current smoking was measured by asking young people how often they smoked tobacco at present, with five response options: ‘I do not want to answer’; ‘I do not smoke’; ‘Less than once a week’; ‘At least once a week, but not every day’ and ‘Every day’. Young people were categorised as non-smokers, occasional smokers and weekly smokers.

### Alcohol use per drinking session

Alcohol use per drinking session was measured by asking young people ‘How many drinks containing alcohol do you have on a typical day when you are drinking?’ with response options of; ‘I never drink alcohol’; ‘Less than one drink’; ‘1 drink’; ‘2 drinks’; ‘3 drinks’; ‘4 drinks’; ‘5 or more drinks’ and ‘I do not want to answer’. These options were combined to produce a three-category ‘alcohol use per occasion’ variable (none, 1–4 and 5+). ‘Binge drinking’ was defined as five or more drinks per drinking session.

### Prevalence and frequency of cannabis use

Students were asked; ‘Have you ever taken cannabis in the last 30 days?’. They were then asked to indicate frequency of use with response options of; ‘Never’; ‘1–2 days’; ‘3–5 days’; ‘6–9 days’; ‘10–19 days’; ‘20–29 days’; ‘30 days or more’ and ‘I do not want to answer’. For analyses, a three-category past-month cannabis use variable was also examined (never, less than daily and daily) as a marker of current use.

### Mephedrone use

Students were asked ‘In your life have you ever tried mephedrone (also called ‘m-cat’ and ‘meow meow’)?’ with response options of; ‘Yes’; ‘No’; and ‘I do not want to answer’. For analyses, a binary variable (‘mephedrone ever use’) was produced.

### Subjective well-being

To measure subjective well-being, students were asked to indicate on a scale of 0–10 how satisfied they were with their life, with a score of 0 indicating the ‘worst possible life’ and 10 the ‘best possible life’.

### Relationships with teachers and peers

Students were asked on a 5-point Likert scale (strongly agree to strongly disagree) whether they felt (1) that their teachers cared about them as a person and (2) that teachers at their school took pupils' ideas seriously. Both items were highly correlated (r=0.63) so summed to form a single item. Students were also asked the extent to which they agreed that they could count on their friends on a 7-point scale from strongly agree to strongly disagree. Finally, students were asked how often they had been bullied in the past couple of months in school (never, once or twice, two or three times, 2–3 times a month, weekly or several times a week).

### Romantic relationships and dating violence

Young people were asked if they had ever been ‘seeing someone’. Those who said yes were asked if a partner had ever (1) made hurtful comments about them, (2) pushed, shoved or slapped them or (3) punched, kicked or beaten them up. Response options were never, once or twice, two or three times, 2–3 times a month, weekly or several times a week. When subjected to factor analysis, these items all indicated loadings of >0.5 on a single factor, and were hence summed to form a total ‘dating violence’ scale.

### Socio-demographic characteristics

Students indicated their sex, year and month of birth. To measure SES, participants completed the Family Affluence Scale (FAS).^70^ The FAS comprises of measures of bedroom occupancy, car and computer ownership, family holidays, dishwashers and bathrooms. These were summed to give an overall measure of family affluence. Ethnicity was asked using the following self-report categories: white, mixed race, Asian or Asian British, black or black British and Chinese or other, and collapsed into a binary ‘white’ and ‘other’ variable.

### Consent

Schools returned a registration form indicating their intention to participate in the study. Schools informed parents about the survey using two of three methods (letters sent home with students or via email, and a text message notification about the letter) and parents had the option of withdrawing their child from data collection (‘opt-out’ consent procedure). The survey was voluntary and completed anonymously. The first question asked students for their consent to participate and if they said no, the survey automatically closed. Schools were provided with information and slides to share with students in advance of the survey.

### Statistical analyses

Reponses where the pupil did not wish to answer were excluded from analyses. Percentages for each level of substance use, life satisfaction and relationship quality are presented, broken down by living arrangements. Percentages are weighted to account for over-representation of minority ethnic groups within the sample. For ease of interpretation of subsequent regression analyses, the living arrangements variable was collapsed into a binary ‘non-foster care’ (ie, not living in FC, and living with mother, father or both) versus ‘foster care’ variable. Regression analyses were then used to examine the association of living in FC with substance use, relationship variables and life satisfaction. Owing to the small number of FC and the low prevalence of substance use, in order to maximise power, substance use variables were condensed into binary variables and subjected to binary logistic regression analysis. Subsequently, a combined substance use variable was created scored 0–3, indicating the number of risk factors (weekly smoking, binge drinking and cannabis use in past month) and subjected to ordinal regression analyses. For teacher relationship quality, ability to count on friends and life satisfaction, frequencies indicated substantial polarisation, with young people in FC at the higher or lower end of the distribution by comparison with peers. Hence, for these variables, multinomial regression models were constructed with three ordinal variables, with the middle category set as the reference. As associations for bullying were linear, the odds of being bullied were analysed using ordinal logistic regression. All models were adjusted for clustering at the school level, and adjusted for age, sex, ethnicity and SES. Finally, all models were rerun with the addition of relationship variables as mediators, to examine the extent to which associations of FC with these outcomes were attenuated by relationship variables. Following the hypothesis that the association of FC with substance use and well-being is partially explained by poorer quality interpersonal relationships, it was anticipated that the ORs and risk ratios for the FC would fall in the second set of models by comparison with the first.

## Results

### Sample characteristics

A total of 87 schools (77% of the 113 network schools) took part in the 2015 SHRN survey. Mean free school meal (FSM) entitlement (a marker of socioeconomic deprivation) was 16.9% within these 87 schools (national average =17.8%). Schools were also representative of schools in Wales according to academic attainment and school size. Thirty-six parents and 1137 children opted out of the survey. We did not capture information on the demographics of this group. A total of 32 479 young people within the eligible age range (11–16 years) completed the survey. Young people who provided sufficient data to categorise their current living arrangements were included in the analyses (88.8%; N=28 838). Optional questions on drug use were completed within 76 of the 87 participating schools; there were no significant differences between schools which did or did not complete these items in terms of socio-demographic characteristics. Socio-demographic characteristics, and substance use outcomes, among young people included in analyses are presented in table 1.

**Table 1:** Sample characteristics

	Frequency (%)
Female	15 200 (52.7)
Age	13.6 (1.4)*
Family Affluence Scale	14.9 (2.0)*
Living arrangements	
Both parents	18 691 (64.8)
Parent and stepparent	3778 (13.1)
Single mother	5323 (18.5)
Single father	747 (2.6)
Foster care	295 (1.0)
Smoking	
Occasional	409 (1.4)
Weekly or more	841 (3.0)
Cannabis use	
Less than daily	468 (1.9)
Daily	167 (0.7)
Alcohol	
1–5 drinks per occasion	6054 (21.6)
5+ drinks per occasion	1690 (6.0)
Ever used mephedrone	261 (1.0)

*Mean (and SD).

### Substance use, life satisfaction and relationship quality by living arrangements

By comparison with young people living in private households, young people in FC reported higher rates of weekly smoking, binge drinking and recent cannabis use (see table 2). Young people in FC reported almost eight times the rates of weekly smoking by comparison with young people living with both parents, and almost four times higher than among those living with a single mother. Young people in FC were more likely to report ever use of mephedrone. In relation to use of multiple hazardous substances, young people in FC were several times more likely than any other group to report simultaneously being regular smokers, cannabis users and binge drinkers, and substantially less likely to report no usage. Young people in FC were also more likely than those in other living arrangements to report poorer relationships with teachers and friends, to have been bullied at least once and to have experienced dating violence. Young people in FC were also more likely to report low life satisfaction. The relationship with life satisfaction was not linear; young people in FC were more likely than any other group to report low life satisfaction, although only young people from two parent families were more likely to report high life satisfaction than those in FC.

**Table 2:** Number (and weighted prevalence*) of young people reporting substance use, life satisfaction and relationship quality outcomes by family structure

	Both birth parents	Single mother	Single father	Parent and stepparent	Foster parents
Smoking status					
None	18 003 (97.1)	4898 (93.6)	664 (90.8)	3481 (93.4)	232 (83.3)
Occasional	207 (1.1)	104 (2.0)	21 (2.9)	70 (1.9)	7 (2.0)
Weekly	333 (1.8)	232 (4.4)	46 (6.3)	182 (4.7)	48 (14.8)
Cannabis use last 30 days					
Never	16 172 (98.3)	4413 (96.8)	616 (95.7)	3133 (95.9)	226 (89.6)
1–29 days	222 (1.4)	108 (2.4)	15 (2.4)	107 (3.2)	16 (5.4)
Daily	62 (0.4)	42 (0.8)	14 (1.9)	33 (1.0)	16 (5.0)
Alcohol use per occasion					
Never	13 781 (74.9)	3547 (68.6)	498 (69.5)	2303 (63.1)	193 (69.4)
1–4 drinks	3661 (20.5)	1169 (23.1)	149 (21.4)	1025 (28.0)	50 (18.2)
5+ drinks	835 (4.6)	421 (8.2)	66 (9.1)	329 (8.9)	39 (12.4)
Mephedrone					
Never	16 269 (99.3)	4523 (98.9)	637 (98.4)	3239 (98.6)	224 (90.0)
At least once	118 (0.7)	59 (1.2)	12 (1.6)	47 (1.4)	25 (10.0)
Multiple substance use index					
0	15 140 (94.3)	3945 (90.0)	542 (87.8)	2798 (89.0)	193 (80.5)
1	661 (4.2)	305 (7.0)	50 (8.2)	223 (7.1)	21 (8.7)
2	166 (1.0)	79 (1.8)	13 (2.2)	74 (2.3)	10 (4.0)
3	82 (0.5)	57 (1.3)	13 (1.8)	10 (1.7)	21 (6.7)
Subjective well-being					
Low	3830 (20.7)	1645 (31.6)	246 (33.8)	1110 (30.2)	101 (35.0)
Medium	7918 (43.3)	2095 (40.7)	306 (41.5)	1573 (43.0)	96 (34.1)
High	6586 (36.0)	1440 (27.6)	182 (24.7)	995 (26.8)	92 (30.9)
Teacher relationship quality					
Low	2402 (13.2)	896 (17.3)	118 (16.1)	635 (17.1)	60 (20.6)
Medium	8414 (46.5)	2322 (45.4)	357 (50.8)	1748 (48.0)	107 (39.8)
High	7287 (40.3)	1901 (37.3)	237 (33.1)	1274 (35.0)	108 (39.6)
Ability to count on friends					
Low	4991 (26.9)	1619 (30.8)	227 (30.8)	1040 (27.9)	103 (36.4)
Medium	6226 (34.1)	1589 (30.8)	238 (32.8)	1148 (31.1)	65 (23.7)
High	7121 (39.0)	1996 (38.4)	266 (36.4)	1524 (41.0)	114 (39.9)
Been bullied					
Never	12 097 (67.6)	3155 (62.6)	442 (63.2)	2104 (58.6)	133 (50.9)
Once or twice	5070 (28.5)	1575 (31.5)	206 (29.3)	1241 (34.8)	104 (39.3)
> twice a month	713 (4.0)	304 (6.0)	53 (7.5)	247 (6.8)	28 (9.7)
Dating violence					
Never	14 983 (85.4)	3955 (79.1)	549 (79.7)	2717 (76.7)	197 (76.5)
At least once	2544 (14.6)	1034 (20.9)	138 (20.3)	836 (23.3)	64 (23.5)

*In some instances, due to rounding, proportions do not add up to 100%.

Regression analyses, comparing young people in FC with participants in other living circumstances (ie, those in dual parent, single parent and stepfamilies), indicated that those in FC were significantly more likely to be regular users of tobacco, alcohol and cannabis, and to have tried mephedrone (table 3; Model 1). For example, the odds of reporting weekly smoking were almost six times higher for young people in FC. Young people in FC were also significantly more likely than young people living with at least one parent to report having been bullied and having experienced dating violence. For quality of relationships with friends and teachers, and for subjective well-being, FC were more likely to report low scores (ie, poor relationships) though simultaneously no less likely to report high scores.

**Table 3:** ORs (and 95% CIs) from logistic regression models, for substance use, life satisfaction and relationship outcomes

			Independent variables					
			Model 1: ORs* (95% CI) Foster care	Model 2: ORs† (95% CI)				
Dependent variables	Sample N			Foster care	Dating violence	Count on friends	Been bullied	Teacher relationships
Smoking status	23977	Non	REF	REF	REF	REF	REF	REF
		Weekly	**5.58 (3.73 to 8.35)**	**4.27 (2.75 to 6.63)**	**1.41 (1.34 to 1.48)**	1.01 (0.96 to 1.07)	**1.14 (1.07 to 1.21)**	**0.82 (0.78 to 0.87)**
Cannabis use last 30 days	21205	Never	REF	REF	REF	REF	REF	REF
		>=once	**4.80 (2.95 to 7.81)**	**3.42 (2.02 to 5.80)**	**1.45 (1.38 to 1.53)**	1.03 (0.98 to 1.09)	1.03 (0.94 to 1.12)	**0.78 (0.73 to 0.83)**
Binge drinking	23675	Never	REF	REF	REF	REF	REF	REF
		Ever	**2.50 (1.54 to 4.06)**	**1.87 (1.11 to 3.15)**	**1.42 (1.37 to 1.48)**	**1.04 (1.00 to 1.09)**	**1.05 (1.00 to 1.11)**	**0.82 (0.78 to 0.85)**
Multiple substance use	20722		**3.72 (2.30 to 6.00)**	**3.10 (1.95 to 4.94)**	**1.47 (1.41 to 1.53)**	1.02 (0.98 **to** 1.06)	**1.06 (1.01 to 1.12)**	**0.80 (0.77 to 0.83)**
Mephedrone	21213	Never	REF	REF	REF	REF	REF	REF
		Ever	**9.24 (5.60 to 15.34)**	**6.35 (3.48 to 11.59)**	**1.45 (1.36 to 1.56)**	0.93 (0.85 to 1.01)	1.08 (0.94 to 1.24)	**0.87 (0.78 to 0.97)**
Subjective well-being	23818	Low	**†1.72 (1.20 to 2.46)**	†1.41 (0.94 to 2.12)	**†1.13 (1.09 to 1.16)**	**†0.89 (0.87 to 0.90)**	**†1.34 (1.30 to 1.38)**	**†0.87 (0.85 to 0.89)**
		Medium	REF	REF	REF	REF	REF	REF
		High	†0.99 (0.68 to 1.46)	†1.02 (0.70 to 1.50)	†0.98 (0.94 to 1.02)	**†1.07 (1.06 to 1.09)**	**†0.89 (0.86 to 0.93)**	**†1.14 (1.12 to 1.17)**
Teacher relationship quality	26496	Low	**†1.83 (1.31 to 2.56)**	–	–	–	–	–
		Medium	REF	–	–	–	–	–
		High	**†**1.22 (0.93 to 1.62)	–	–	–	–	–
Ability to count on friends	26837	Low	**†1.88 (1.23 to 2.88)**	–	–	–	–	–
		Medium	REF	–	–	–	–	–
		High	**†1.53 (1.03 to 2.28)**	–	–	–	–	–
Been bullied	26168		**1.80 (1.38 to 2.35)**	–	–	–	–	–
Dating violence	25742	Never	REF	–	–	–	–	–
		Ever	**1.66 (1.13 to 2.43)**	–	–	–	–	–

Bold typeface signifies significant results.

†Indicates relative risk ratios (RRR) rather than ORs.

*All models were adjusted for clustering at the school level, and adjusted for age, sex, ethnicity and SES—model 1 comprises the foster care variable, model 2 comprises the foster care variable and all relationship variables.

As indicated in Model 2, where relationship variables are included in all models, experiences of dating violence and quality of teacher relationships were significantly associated with substance use outcomes and life satisfaction. Reports of having been bullied were significantly associated with all variables, other than cannabis use. Ability to count on friends was associated only with life satisfaction and with binge drinking. However, associations operated in inverse directions; young people who reported being better able to count on friends reported poorer life satisfaction, but were more likely to be binge drinkers. Once associations of relationship variables are accounted for in final models, the association of FC with subjective well-being was no longer statistically significant. In these models, there remains a significant association of FC for all substance use variables, but the association of FC with all outcomes is attenuated, in most cases by 20–30%. Hence, the analyses are consistent with a hypothesis that the associations of living in FC with substance use and life satisfaction are partially explaining by poorer quality social relationships.

## Discussion

### Present findings and previous literature

Consistent with international literature, this paper found that young people in FC in Wales had higher rates of use of substances (ie, tobacco, cannabis use, binge drinking and mephedrone) and poorer subjective well-being than those from private households.^2–13^ Second, and again consistent with international literature demonstrating increased relationship difficulties among FC including attachment disorders^49–51^ and increased exposure to bullying,^71^ ^72^ young people in FC in Wales reported poorer relationships with peers and teachers, and experienced higher rates of bullying and dating violence. Third, consistent with a hypothesis that good quality relationships may be protective against poorer health outcomes,^36–41^ relationships with teachers, experiences of bullying and dating violence were all significantly correlated with increased risk of substance use and poorer subjective well-being. A review^73^ commissioned by the National Institute for Health and Clinical Excellence (NICE) and the Social Care Institute for Excellence (SCIE) identified and synthesised the results of 50 studies on the views, experiences and preferences of children in care, and physical, emotional, social and other outcomes important to them. Nine major outcomes were identified, of which several were related to positive, secure relationships: love; a sense of belonging; being supported, with emphasis on emotional and educational support; having someone to talk to and stigma and prejudice associated with being in care. Unmet needs for love and affection were perceived by some children in care to have a significant and lasting impact on outcomes in later life.

Associations of peer relationships were, however, more variable; while a higher perceived ability to count on friends was associated with significantly improved subjective well-being, it was not associated with most substance use outcomes. The clearest association was for alcohol, with a higher ability to count on friends associated with a higher risk of binge drinking. This is perhaps unsurprising given an extant literature demonstrating the roles of alcohol in social bonding and friendship formation, and the socially contagious nature of substance use during adolescence.^74^ ^75^ Although beyond the scope of the present study, it is plausible that LAYP may be particularly susceptible to peer influence, due to instability of relationships with primary caregivers, and hence friendships may, to some extent, exacerbate substance use rates.

To the best of our knowledge, this study is the first to examine the role of social relationships experienced by LAYP in attenuating associations with substance use and subjective well-being. After adjusting for relationship variables, the association of FC with subjective well-being diminished to below significance. Although there remained a significant association of FC for all substance use variables, ORs for all outcomes were reduced substantially. Hence, the findings are consistent with a hypothesis that poorer substance use and subjective well-being outcomes for LAYP are partly explained by difficulties in social relationships.

### Limitations and future research

The cross-sectional design of this study means that causality cannot be established. Notably, the research team plans to include unique identifiers in future rounds of the survey, which will enable longitudinal analyses of trajectories in care among secondary school-aged children. The sensitivity of some of the topics, for example, intimate partner violence and substance use, may have led to under-reporting. The paper involved secondary analyses of a data set not designed explicitly for this purpose, and while most of the measures are adopted from previous surveys, some (such as measures of novel psycho-active substances) have not been widely used previously. All models were adjusted for SES; however, we are uncertain of the validity of this measure, based on family affluence, for use among young people in FC since they have been exposed to more than one household. The indicator for living in care was a crude measure of care status. The survey question may have been confusing for children living at home under placement with parent regulations, or for children living in kinship care, neither of which may have acknowledged they were under care orders. This may have led to children with care experiences being in the comparator group. We measured only current FC status rather than histories of care: the survey did not assess duration and number of placements, which have been associated with poorer outcomes among young people in FC.^17^–^16^ For example, more stable placements for LAYP have been found to be protective of dating and relationship violence.^54^ We did not examine differences in outcomes among different types of care placements, for example, residential care versus foster care. It is possible that children living in residential care or residential schools were excluded from the foster care group. To date, the literature on differences in outcomes among children in different types of care is scarce. The sensitivity of the care variables will be explored further in future rounds of the survey with more detailed measures of care status, for example, having ever been in care, placement type and length and number of placements. Arguably, the impact of early attachment instability on subsequent relationships is not limited to a particular relationship type.^48^ However, the survey also measured a limited range of social relationships; for example, we did not examine the perceived quality of other important relationships which may differ by living arrangements and contribute to substance use and well-being, such as relationships with the birth parents or foster families.

Nevertheless, the study signals some important future directions for research. There remains a range of potential mechanisms underlying the associations observed, which could be usefully tested in future research. For example, disentangling the contribution of dimensions of the care experience to outcomes, such as placement instability, from precare adverse experiences, is an important future direction. The development of more high-quality data infrastructure for monitoring outcomes among young people in care longitudinally is an important potential future direction. At present, national cohorts are incomplete,^76^ and retrospective studies often rely on poor quality routine data.^77^ Subsequent rounds of SHRN data collection, involving data from larger samples and refined questions, may enable more detailed analyses of the extent to which observed associations remain after adjusting for experiences of care itself, or stem from experiences which preceded entry to foster care. Data linkage has been used extensively to facilitate research on outcomes among children in care in Sweden,^78^ and linkage with other datasets here in Wales and the rest of the UK, including the CIN census,^25^ the Patient Episode Database for Wales (PEDW),^79^ and general practice datasets,^80^ if achieved, will facilitate longitudinal analyses of trajectories in and out of the care system and their impacts on health and well-being.

### Implications for policy and practice

The study has important implications for policy and practice. First, intervention approaches to improve well-being and reduce substance use among young people in foster care should include a focus on supporting LAYP in the development of healthy interpersonal relationships. This may, for example, require appropriate therapeutic interventions such as counselling and other psychological therapies on entering care in order to mitigate the effects of precare ACEs on the formation of future relationships. School-based interventions to reduce bullying, promote healthy romantic relationships and positive teacher–student relationships in the school setting may play an important role in mitigating the tendency for already disadvantaged young people experiencing further difficulties in social relationships with teachers, peers and romantic partners. Finally, changes to service and research infrastructure, including data collection, sharing and analyses systems among local authorities, hospitals, general practitioners and other services that come into contact with young people in care are required to facilitate large-scale interdisciplinary studies that can provide a mechanism for outlining epidemiology, and for monitoring and informing new and existing interventions.
